# Direct evidence for encoding of motion streaks in human visual cortex

**DOI:** 10.1098/rspb.2012.2339

**Published:** 2013-02-07

**Authors:** Deborah Apthorp, D. Samuel Schwarzkopf, Christian Kaul, Bahador Bahrami, David Alais, Geraint Rees

**Affiliations:** 1 School of Psychology, University of Wollongong, Wollongong, New South Wales 2522, Australia; 2 Institute of Cognitive Neuroscience, University College London, 17 Queen Square, London WC1N 3AR, UK; 3 Wellcome Trust Centre for Neuroimaging, University College London, 12 Queen Square, London WC1N 3BG 10003, UK; 4 Interacting Minds Project, Institute of Anthropology, Archaeology and Linguistics, Aarhus University, Aarhus University Hospital, Norrebrogade 44, Building 10 G, 8000 Aarhus C, Denmark; 5 Centre of Functionally Integrative Neuroscience, Aarhus University Hospital, Norrebrogade 44, Building 10 G, 8000 Aarhus C, Denmark; 6 School of Psychology, University of Sydney, Sydney, New South Wales 2006, Australia

**Keywords:** motion, visual cortex, motion streaks, multi-voxel pattern analysis

## Abstract

Temporal integration in the visual system causes fast-moving objects to generate static, oriented traces (‘motion streaks’), which could be used to help judge direction of motion. While human psychophysics and single-unit studies in non-human primates are consistent with this hypothesis, direct neural evidence from the human cortex is still lacking. First, we provide psychophysical evidence that faster and slower motions are processed by distinct neural mechanisms: faster motion raised human perceptual thresholds for static orientations parallel to the direction of motion, whereas slower motion raised thresholds for orthogonal orientations. We then used functional magnetic resonance imaging to measure brain activity while human observers viewed either fast (‘streaky’) or slow random dot stimuli moving in different directions, or corresponding static-oriented stimuli. We found that local spatial patterns of brain activity in early retinotopic visual cortex reliably distinguished between static orientations. Critically, a multivariate pattern classifier trained on brain activity evoked by these *static* stimuli could then successfully distinguish the *direction* of fast (‘streaky’) but not slow motion. Thus, signals encoding static-oriented streak information are present in human early visual cortex when viewing fast motion. These experiments show that motion streaks are present in the human visual system for faster motion.

## 1. Introduction

**Figure 1.**
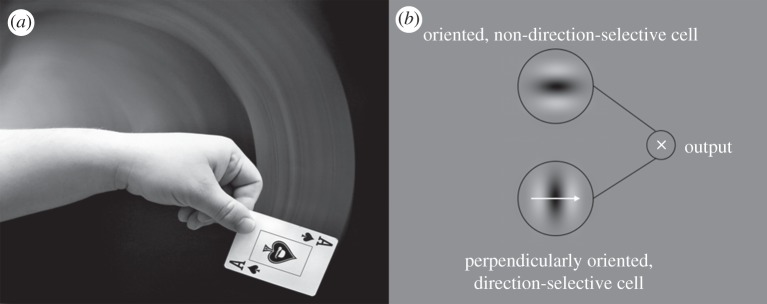
Motion streaks in art and vision. (*a*) Motion streaks are often used in photography and art to give a strong impression of fast motion within a scene. Photograph by Tod Klassy, sourced from www.flickr.com and reproduced with permission. (*b*) Geisler's [1] model of how a motion streak might be combined with a motion signal in early cortex to provide a code for motion direction. Specifically, a direction-selective V1 cell (giving the sign of motion direction) might combine its output with that of a cell selective for static orientation, which would respond to the temporally integrated motion streak, giving fine angular resolution and solving the aperture problem [1].

Blurred lines or ‘motion streaks’ along the trajectory of a moving object have long been used in art and photography to illustrate fast motion (figure 1*a*). More recently, it has been suggested that these streaks, which occur in the visual system due to temporal integration [2], could be used to resolve inherent ambiguities in motion direction perception [1] (figure 1*b*). Specifically, the orientation of a static motion streak carries information about motion direction. Consistent with this, neurons in macaque V1 respond increasingly strongly to orientations parallel to their preferred direction of motion with increasing speed [3], which tallies with other reports of speed-related variations in directional selectivity in these neurons [4–6]. In humans, parallel-oriented noise impairs direction discrimination [7], and ‘streaky’ motion causes effects very similar to those found in the classical orientation literature [8–10]. However, there is hitherto little physiological evidence of any involvement or indeed the presence of streaks in human motion direction perception. In particular, there has not been evidence for motion streaks in retinotopic early visual cortices sensitive to static stimulus orientation.

Most studies of motion in the human visual system implicitly assume processing in early vision by units most sensitive to orientations *orthogonal* to their preferred directions, and that networks of these units pass their output to higher motion areas such as hMT+/V5, which is strongly responsive to motion compared with static stimuli [11–13]. However, if orientation-selective mechanisms *parallel* to motion direction (i.e. motion streaks) in early human visual processing (e.g. V1, V2) contribute to the perception of motion [1] (figure 1), then these early neuronal populations should show selectivity for static-oriented stimuli *parallel* to the direction of motion. Psychophysical adaptation studies assume that neurons sharing selectivity for a stimulus will show reduced response to a subsequently presented stimulus that is detected by the same population of neurons. Thus, we hypothesized that if faster motion is more likely to be detected by neurons tuned to parallel orientations, then adapting to faster motion should raise detection thresholds for parallel static orientations. Meanwhile, multi-voxel pattern analysis (MVPA) can reveal selectivities for orientation and direction of motion of visual stimuli from population fMRI responses by exploiting information contained by the spatial pattern of signals in a brain region [14–16].

To test whether these distinct neural effects of faster and slower motion existed in the human brain, we first performed a psychophysical adaptation study testing contrast threshold elevation for static-oriented patterns after viewing fast or slow motion. To anticipate our findings, after prolonged adaptation to faster motion, thresholds were elevated more for patterns *parallel to motion direction*, but, crucially, adaptation to slower motion elevated thresholds for *orthogonal* patterns. In a second experiment, using fMRI MVPA, we then investigated whether ‘motion streaks’ contributed to motion processing in the human brain by testing whether activity patterns in neuronal populations selective for orthogonal *static* orientations might be sufficient to determine the direction of motion of faster (but not slower) *moving* stimuli that produced ‘motion streaks’ with the same orientations (and vice versa). To test this, we measured activity in retinotopic cortical areas V1–V3 and V5/hMT+ during perception of static-oriented stimuli as well as faster- and slower-moving stimuli. We found that a classifier trained on patterns of brain activity while viewing static-oriented stimuli could successfully decode the direction of dot stimuli moving fast enough to form streaks, but not those moving at speeds below the streak threshold.

## 2. Methods

### (a) Experiment 1: psychophysics

#### (i) Participants

Eight experienced psychophysical observers (three female), aged between 27 and 46 years, all of whom had normal or corrected-to-normal vision, gave informed consent to participate in the experiment that was approved by the local ethics committee. Two were authors and the other six were naive to the purpose of the experiment.

#### (ii) Apparatus

Stimuli were programmed in Matlab (v. 7.4), using the Psychophysics Toolbox [17,18]. Participants viewed the stimuli on a Sony Trinitron multiscan G500 22″ CRT monitor with a screen resolution set to 1024 × 768 pixels and a vertical refresh rate of 100 Hz, controlled by a Mac Pro computer with a dual-core Intel Xeon processor. A Cambridge Research Systems Bits++ digital-to-analogue converter was used to provide 14-bit resolution in order to enable precise measurement of low-contrast thresholds. The monitor was gamma-corrected in the software to achieve linearity of output. Observers viewed all stimuli from a distance of 57 cm.

#### (iii) Stimuli and procedure

**Figure 2.**
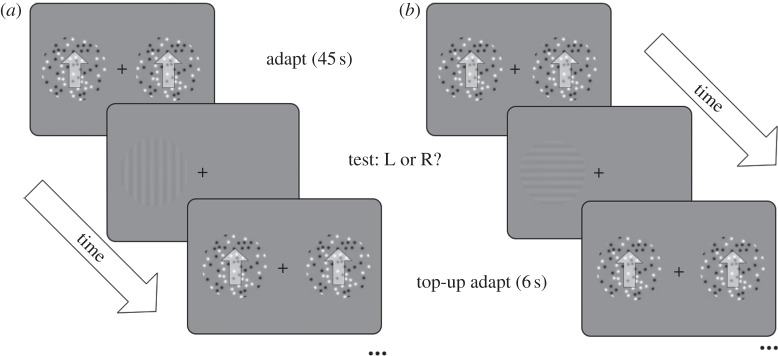
Schematic of the procedure for the psychophysical experiment. Participants adapted to vertical motion, either faster (13 m s^−1^) or slower (1.6 m s^−1^) than the streak threshold for dots of this size. They were then asked to detect a low-contrast grating, either parallel (*a*) or orthogonal (*b*) to the direction of motion, which appeared either to the left or to the right of fixation, in the same retinal location as the adapting dots. Contrast of the test grating was controlled by a QUEST adaptive staircase; see §2 for full details.

Participants viewed the stimuli binocularly, using a standard chinrest. Adapting stimuli were composed of two drifting random dot displays, each composed of 80 Gaussian blobs with a standard deviation (s.d.) of 0.08°, giving a dot diameter (defined as 4 × dot s.d.) of 0.32°. Half of the dots were dark and half were light, drifting with 100 per cent coherence on a mid-grey background. Maximum and minimum dot luminances were 67.3 and 0.26 cd m^−2^, and background luminance was 33.8 cd m^−2^. Faster dots drifted at 13.02° s^−1^, whereas slow dots drifted at 1.63° s^−1^. Respectively, these speeds were well above and well below the speed of dot motion purported to be critical to the generation of motion streaks, known as Geisler's critical streak speed [1,10]. Dot speed was controlled by manipulating the pixel step size for each video frame. The procedure is illustrated in figure 2. Adapting dots were presented in two virtual circular apertures 4.88° in diameter, 3.81° to the left and right of a white fixation cross, and always drifted directly upwards. During the test phase, the fixation cross-changed to black and the test stimulus (a low-contrast sine wave grating with a spatial frequency of 1.54 cycle per degree) appeared briefly either in the left or in the right aperture. The subject's task was to indicate whether the low-contrast test grating appeared in the left or right aperture. Subjects initially adapted for 42 s to the motion stimuli, 200 ms after which the test grating appeared for 10 ms and the subject keyed their response (‘left’ or ‘right’). Subsequent trials involved 6 s of top-up adaptation. Test stimuli were either parallel or orthogonal to the direction of motion, in separate blocks. Contrast of the test stimulus was manipulated in two interleaved adaptive staircases using the QUEST procedure [19] to determine subjects' contrast thresholds for grating detection after adaptation. In a control condition, unadapted thresholds were obtained by removing the adapting dots. Threshold elevation was measured in decibels, as given by equation (2.1):2.1
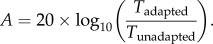

### (b) Experiment 2: fMRI pattern classification

#### (i) Participants and experimental design

Eight neurologically healthy adult volunteers (three females) with normal or corrected-to-normal vision aged between 25 and 42 years gave informed consent to participate in this study. All procedures were approved by the local ethics committee. Stimuli were generated in Matlab (v. 7.4, Mathworks) and presented in the scanner on a NEC LT158 data projector and viewed on a mirror mounted on the head coil. There were six different stimulus conditions (three stimulus types: static, slow, fast × two orientations/directions: 45° or 135°). Each condition occurred once within each run, presented in a randomized block design (22.4 s block duration), interleaved with 16 s blank fixation blocks (figure 4*a*). Overall, 10 runs were presented per participant. Participants viewed the stimuli while performing a fixation-dimming task. All stimuli were presented within a circular annulus, softened at the edges with a cosine ramp, with an inner radius of 2° and an outer radius of 8° (figure 4*a*); an additional 1° gap was added along the vertical midline to assist in localizing the borders of V1 and V2. Motion stimuli were 250 black and white Gaussian blobs, each with a standard deviation of 0.14°, giving a nominal ‘dot width’ (4 × dot s.d.) of 0.55°. Dots moved at either 11.3° per second (fast) or 2.3° per second (slow), either upwards to the left (135°) or upwards to the right (45°; figure 4*a*). We used these orientations rather than vertical and horizontal motion/orientation to avoid the well-known horizontal bias [20,21], which could have artificially elevated the classification accuracies. Speeds were slightly closer together than the speeds in the psychophysical experiment to maximize the possibility of finding similarities between faster and slower motions while remaining on either side of the critical streak speed. Oriented stimuli were composed of randomly generated noise stimuli, filtered in the Fourier domain in both orientation and spatial frequency to give orientations of 45° and 135°, with a one-octave bandwidth of spatial frequencies centred around 1.36 cycle per degree (figure 4*a*), and a 7.5° bandwidth of orientations. Stimuli were presented in 750 ms intervals, followed by a 250 ms blank period. Motion and orientation stimuli were randomly generated for each interval, to avoid local contrast cues biasing the results.

#### (ii) Display checks

Prior to the experiment, we determined whether there was any temporal blurring on the display that might cause actual streaks, because LCD projectors can show sluggish response times. To characterize the response time of the projector in the most accurate manner possible, we measured the luminance from the projector with a photodiode (sampling rate: 500 Hz) inside the scanner, using the actual luminance values to be presented during the experiment. We measured the response time as the display changed from grey to black, black to grey, grey to white and white to grey. None of these response times exceeded 4 ms, and thus we were confident that motion blur artefacts on the screen would not be present during the experiment.

#### (iii) fMRI acquisition

Functional data were acquired on a Siemens Allegra 3T MRI scanner, using a standard transmit/receive single-channel (birdcage) head coil with a single-shot gradient echo isotropic high-resolution EPI sequence (matrix size: 128 × 128; FOV: 192 × 192 mm^2^; in-plane resolution: 1.5 × 1.5 mm^2^; 32 oblique transverse slices with interleaved acquisition; slice thickness: 1.5 mm, no inter-slice gap; TE: 30 ms; acquisition time per slice: 100 ms; TR: 3200 ms; echo spacing: 560 μs; receiver bandwidth: 250 kHz; 30% ramp sampling; twofold read oversampling to allow for k-space re-gridding; read gradient amplitude: 34.47 mT m^−1^; read gradient slew rate: 344.7 mT m^−1^ ms^−1^; flip angle *α* = 90°). Slices were angled at 30° to maximize coverage of the calcarine sulcus and the occipital lobes. Seventy-seven images were acquired in each run of the main experiment. In addition, we also acquired T1-weighted structural images for each participant. Further, we acquired two runs of retinotopic mapping. Each session comprised five alternating blocks of 10 volumes stimulating the vertical and horizontal meridians with flickering checkerboard wedges (frequency = 6.2 Hz, horizontal diameter = 13°); vertical diameter = 11° followed by rest (grey background) blocks of six images. Finally, we also acquired runs of a motion localizer showing random dot stimuli comprising black and white dots on a grey background presented within a circular aperture around fixation (diameter: 11.5°). In alternating blocks of six images, we showed either translating motion (half of the dots translated in opposite directions) or static dot stimuli. For the motion stimuli, the direction changed at random every 800 ms (all directions from 0° to 345° with 15° increments). For the static stimuli, a new random dot stimulus was presented every 800 ms.

#### (iv) Initial data analysis

Data were analysed using SPM5 (http://www.fil.ion.ucl.ac.uk/spm/). We discarded the first five images of each scanning run to allow for magnetic saturation. After this, images were realigned and coregistered to the individual structural scans for each participant, and data were spatially smoothed using a 4 mm full width at half maximum (FWHM) kernel. For the second-level group analysis, images were spatially normalized to the MNI template. For the univariate analysis and the localizer sessions, a general linear model was fitted to the data using regressors for each of the experimental conditions and covariates of no interest for the motion parameters. Regions of interest were delineated manually using software Freesurfer (http://surfer.nmr.mgh.harvard.edu) by first segmenting the structural scan for each participant and reconstructing an inflated mesh of the boundary between grey and white matter to project the activations from the localizer runs onto this surface. For retinotopic mapping, we contrasted the response to vertical and horizontal meridian stimulation, and drew the boundaries of areas V1–V3 along the peaks of the positive and negative activations. We defined V5/hMT+ by contrasting the response to moving and static stimuli and selecting the cluster of significant voxels in lateral occipital cortex. A control region, where above-chance decoding would not be expected, was defined in frontal cortex for each participant in an area that showed no stimulus-specific activity, as defined by contrasting the response to all conditions with fixation rest blocks. Binary volume masks for each region of interest (ROI) were then generated by projecting the grey matter voxels that fell within a region back from the surface into the native volume for each participant.

#### (v) Multi-voxel pattern decoding

We first normalized the data from each run by calculating the *z*-score for each voxel across the time series from each run. Subsequently, we averaged the images from each stimulus block after shifting the time series by 1 TR (3.2 s) to account for haemodynamic lag. To decode the orientation/direction, we extracted the voxels from each ROI for each block average and vectorized them. These vectors constituted the pattern of voxel activity for each stimulus block. A class label was assigned to each pattern to indicate whether its orientation/direction was 45° or 135°, and from which of the three stimulus types (static, slow-moving, fast-moving) it originated. We used a standard leave-one-run-out cross-validation procedure for decoding. Briefly, we trained a linear support vector machine [22,23] to distinguish the orientation/direction labels of voxel patterns from nine out of the 10 runs, and subsequently tested whether the algorithm could classify the labels of voxel patterns in the final, independent test run. This procedure was repeated using each of the 10 runs as test data. Decoding performance for each participant was then calculated as the proportion of classifications across all cross-validations in which the test labels were assigned correctly. Consistent with previous reports [24,25], qualitatively similar results were obtained when using other classifiers (pattern-correlation and linear discriminant analysis; see electronic supplementary material for details).

We tested for successful decoding in a region by testing whether decoding performance was significantly different from chance using a statistical threshold of *p* < 0.05 corrected by the number of ROIs tested (i.e. *p* < 0.01). While we had a prior hypothesis of successful decoding in motion streak information in early visual cortex, this was necessary as the number of comparisons could otherwise have inflated false-positive rates.

To further support any findings of decoding significantly above-chance levels, we also conducted a permutation analysis to estimate the breadth of the distribution of decoding accuracies that could be expected by chance. In 50 000 independent iterations, we generated a simulated data sample under the exact conditions as in the experiment (i.e. eight participants, 10 runs, two trials per run) but where the probability of correct decoding in each trial was 0.5. This determined that the 95% CI of the chance distribution was between 0.425 and 0.575.

## 3. Results

### (a) Experiment 1: psychophysics

**Figure 3.**
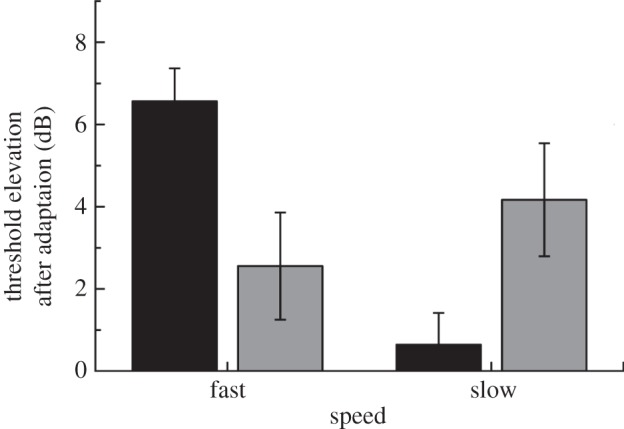
Mean results from the psychophysical adaptation experiment for eight participants. Error bars denote ±1 s.e.m. There was a significant interaction between orientation and speed, *F*_1,7_ = 39.29, *p* < 0.001; see §§3 and 4 for details. Black bars, parallel; grey bars, orthogonal.

First, we tested the psychophysical effects of adapting to faster or slower motion, respectively, on contrast thresholds for detecting-oriented stimuli. We predicted that, if streaks are encoded by the same mechanisms that encode static orientations parallel to the direction of fast motion, then we would subsequently find elevated thresholds for detecting static patterns parallel to the adapting faster motion, relative to orthogonal thresholds. But crucially, if slower motion is encoded by neurons whose preferred orientation is *orthogonal* to their preferred direction [5,26–28], then we expected instead to see the opposite pattern after adaptation to slow motion. Participants adapted for 40 s, followed by 10 s top-up adaptation periods, to stimuli on either side of fixation that were moving in an upwards direction, at either slow or fast speeds (see §2 for details). We measured thresholds for the detection of a low-contrast grating presented either to the left or to the right of fixation, and calculated threshold elevation in decibels (see equation (2.1)) for eight participants. Mean threshold elevations are plotted in figure 3.

Consistent with our hypothesis, we found psychophysical evidence that participants' thresholds for the visibility of static, oriented stimuli were affected differently by adapting to faster (streaky) and slower (non-streaky) motion. Adapting to faster motion caused significantly greater threshold elevations for stimuli parallel to the motion direction, whereas adapting to slower motion caused greater elevation for *orthogonal* stimuli. There was a significant main effect of speed, *F*_1,7_ = 11.55, *p* = 0.011, and a significant interaction between speed and orientation, *F*_1,7_ = 39.29, *p* < 0.001. Importantly, threshold elevation was higher for parallel than for orthogonal gratings after adapting to faster motion (*p* < 0.05, corrected), but after adapting to slower motion, orthogonal thresholds exceeded parallel (*p* < 0.05, corrected).

### (b) Experiment 2: fMRI

#### (i) Univariate analysis

**Figure 4.**
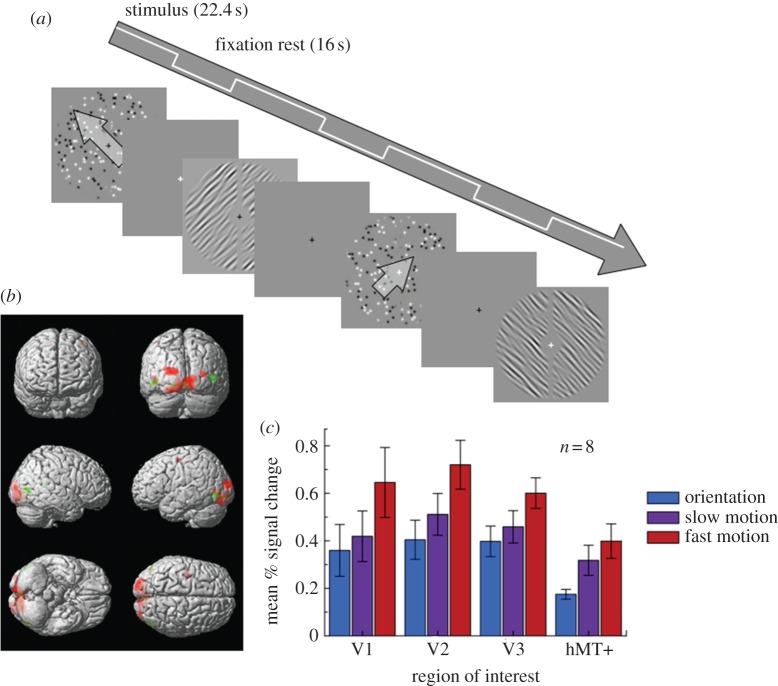
Procedure for the fMRI experiment and univariate results. (*a*) Schematic of the block design within each scanning session. (*b*) Statistical parametric maps from a single representative participant overlaid on a three-dimensional reconstruction of a T1 template brain in the stereotactic space of Talairach & Tournoux [29]. Red colours indicate those cortical loci that showed greater BOLD responses to faster compared with slower motion. A threshold of *p* < 0.001 (uncorrected) is used for display purposes. Green regions showed greater responses to slow motion than oriented stimuli (*p* < 0.001). (*c*) Mean per cent BOLD signal change (relative to global mean) in each region of interest, averaged over eight participants. Error bars denote ±1 s.e.m.

We acquired high-resolution (1.5 × 1.5 × 1.5 mm^3^) blood oxygen-level dependent (BOLD) images from retinotopic cortical areas V1–V3 and V5/hMT+ (localized on a per-participant basis in independent scans) while participants viewed static-oriented stimuli (45° or 135°) or faster (‘streaky’) and slower random dot stimuli moving in corresponding directions (figure 4*a*). Using conventional univariate analyses (see §2), we compared the activation by the three stimulus types in each of these regions. The fMRI response to faster motion was generally stronger. There was a main effect of speed, *F*_2,14_ = 26.1, *p* < 0.001, and also of ROI, *F*_3,21_ = 4.62, *p* = 0.012. There was also a significant interaction between speed and ROI, *F*_6,42_ = 2.839, *p* = 0.021. Strikingly, however, the response in almost all the early visual areas to slower motion was comparable to that to oriented stimuli. Only in V2 did we observe significantly greater responses to slower motion than to oriented stimuli, whereas the response in V5/hMT+ did not differ between the two speeds of motion (see table 1 for more detail on these comparisons). Taken together, these data show that early visual cortex responded more strongly to faster than to slower motion, while V5/hMT+ was activated similarly by both kinds of motion. Motion streaks induced by fast-moving stimuli may thus contribute to the responses of early visual areas (V1–V3) to these stimuli.

**Table 1.** Univariate region of interest (ROI) analysis: *t*-values for paired *t*-tests between motion and orientation conditions in each ROI, averaged over the eight participants. *p*-Values are shown in parentheses, Bonferroni-corrected for multiple comparisons.

	V1	V2	V3	hMt+/V5
fast > slow	3.07 (0.07)	3.98 (0.02)	4.38 (0.01)	2.52 (0.15)
slow > ori.	2.43 (0.18)	4.11 (0.02)	2.30 (0.22)	2.67 (0.12)

It was important to show that changing the orientation (of the static stimuli) or the direction of motion (of the moving stimuli) did not produce differences in activation at the univariate level, as this would render any MVPA redundant. We compared the per cent signal change in each individually defined region for 45° and 135° conditions for fast, slow and static stimuli. There were no significant differences in overall brain activity in any of these areas (all *p*-values > 0.1, corrected; see the electronic supplementary material, for details).

#### (ii) Multi-voxel pattern analysis

We reasoned that if motion streaks were involved in the processing of moving stimuli, then response patterns in neuronal populations selective for *static* orientations should be sufficient to determine the direction of motion of *faster*-*moving* stimuli that produced ‘motion streaks’ at the *same* orientations. At the same time, such static orientation signals should *not* be sufficient to predict the direction of non-streaky motion produced by slower-moving stimuli. We therefore used MVPA, which can decode the orientation and direction of motion of visual stimuli from population fMRI responses by exploiting information contained by the spatial pattern of signals in a brain region [14–16]. We trained a linear support vector machine classifier [22,23] on activity evoked by the stimuli in early retinotopic cortices to decode the direction of motion or the orientation of the different stimulus types (see §2 and electronic supplementary material for details).

**Figure 5.**
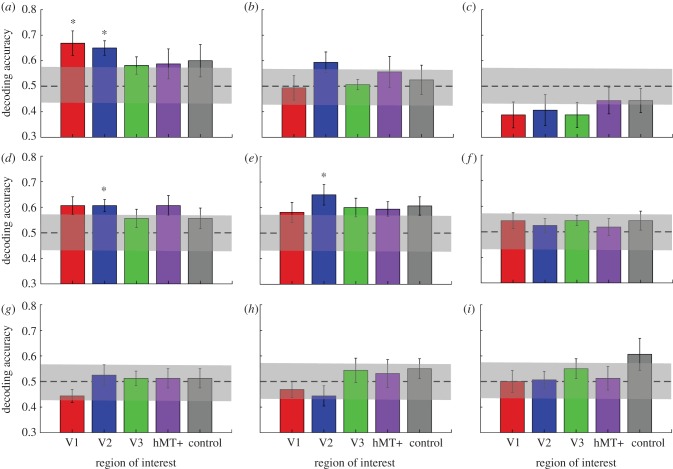
Mean decoding accuracy across eight participants for five regions of interest. (*a*) Results for decoding the orientation of static stimuli (45° versus 135°) for stimulus-responsive regions in early retinotopic visual cortex plus V5/hMT+ and a control region in prefrontal cortex. (*b*,*c*) Results for decoding of faster and slower motion. (*d*,*e*) Training the classifier on discriminating orientation but testing it by discriminating the direction of faster motion (*d*) or vice versa (*e*). (*f*) Training the classifier to discriminate the orientation, but testing it on slower motion. (*g*,*h*) Generalizations for faster-to-slower and slower-to-faster motion. (*i*) Generalization from slower motion to orientation. The dashed line indicates chance performance, and the shaded region indicates its 95% CI (see §2 for details). Error bars denote ±1 s.e.m. Asterisks indicate regions where decoding accuracy was significantly (*p* < 0.01, two-tailed *t*-test) different from chance performance.

We found that spatially distributed response patterns in all of the early retinotopic visual areas V1 (*t*_7_ = 3.51, *p* = 0.0099) and V2 (*t*_7_ = 5.29, *p* = 0.0011) were sufficient to decode the orientation of the static stimuli significantly (*p* < 0.05, corrected for number of ROIs) better than chance (figure 5*a*). However, it was not possible to reliably decode the orientation of the static stimulus from voxel patterns in V3, V5/hMT+ or our control region, a frontal area defined by the absence of any stimulus-specific response (all *p*s > 0.05, corrected for multiple comparisons). This is consistent with previous findings of robust encoding of orientation in the pattern of activity across voxels in early visual cortex [14,16], but not in V5/hMT+ [14]. However, we did not observe significant decoding for the direction of motion for dot stimuli moving at either slower or faster speeds (figure 5*b*,*c*).

We next tested whether classifiers trained in this way on *static*-oriented patterns would generalize to moving patterns, even though the static stimuli contained no motion information (either explicit or implied). We found training classifiers to distinguish static orientations generalized well to decoding the direction of faster motion at a level significantly (*t*_7_ = 4.43, *p* = 0.003) above chance only in area V2 (figure 5*d*). Training classifiers to distinguish faster motion directions and testing on orientation produced very similar results (figure 5*e*). By contrast, we observed no significant generalization from orientation to decoding the direction of slower motion, or vice versa, in any of the regions (figure 5*f*,*i*; all *p*s > 0.05). A repeated-measures ANOVA on the classification accuracies in all visual areas for training on static orientations and testing on faster compared with testing on slower motion (figure 5*d,f*) showed a significant main effect of speed, *F*_1,7_ = 12.003, *p* = 0.01, but no main effect of ROI (*p* = 0.88) and no significant interaction (*p* = 0.43). Finally, we did not observe any significant decoding in the control region in frontal cortex in any of these comparisons (all *p*s > 0.05). To further support these decoding results, we also determined the confidence interval for chance performance (shaded bars in figure 5; see §2 for details). Clearly, only the accuracies for orientation decoding in V1 and V2, as well as the generalization tests between orientation and fast motion in V2, are significantly different from chance.

In summary, our results support the hypothesis that motion streaks caused by faster motion are encoded in retinotopic visual cortex [3] by neural mechanisms selective for static-oriented patterns. Moreover, the absence of successful generalization between the two speeds indicates that the neural representation of direction differed between slow- and fast-moving stimuli. The fact that voxel response patterns produced by static stimuli are informative about the direction of fast motion is consistent with the notion that motion streaks play a role in encoding direction of motion. Moreover, our results were independent of the algorithm used for MVPA, as we found comparable results when we used a simple pattern-correlation classifier (see the electronic supplementary material for details), thus showing that our findings were robust to testing with different classification algorithms [24].

#### (iii) Eye movements and behavioural data

We measured eye movements to check that participants were fixating accurately, and not tracking the motion, which could have led to systematic differences between the conditions. However, no differences between any of the conditions were seen in the eye-movement patterns (see electronic supplementary material for details). We also measured participants' performance on the fixation-dimming task (where they were asked to press a button every time the fixation cross-changed colour), to check for any differences in overall alertness between the conditions. Again, no systematic differences were found (see the electronic supplementary material for details).

## 4. Discussion

Here, we provide both psychophysical and physiological evidence for different processing of faster and slower motions in the human brain. Specifically, our fMRI results, using a conservative correction for multiple comparisons, showed successful generalization from training the decoding of static-oriented stimuli to testing the decoding of direction of faster (but not slower) motion in area V2, whereas our psychophysical results suggest that faster and slower motions may be processed by distinct neural substrates.

Our psychophysical results provide evidence that the neural signature of adaptation to faster and slower motions is quite different; slower motions adapted orthogonal orientations, whereas faster motions adapted parallel orientations. This is in line with single-neuron recording [3] and optical imaging [6] studies, and, unlike previous psychophysical studies, shows a clear dissociation between the effects of faster and slower motion, which implies that slower motion does not merely have less effect owing to a weaker signal. We reasoned that if faster motion was adapting populations of orientation-selective cells in early cortex, then cross-selectivity of these populations might account for previous successful decoding of motion by MVPA in early cortex, but not, paradoxically, in higher motion-selective areas [15,30].

The fMRI findings provide direct evidence for neural correlates of motion streaks in *early* human retinotopic visual cortex. Although previous work [31] has shown that human V5/hMT+ responds to coherent Glass patterns, consistent with the motion streak hypothesis, earlier visual areas (where streaks are thought to be formed) have not previously been explored. It should be pointed out that recent research on the human motion complex [32,33] reveals that human motion-processing areas are less analogous to monkey MT than previously assumed, and that the area designated as hMT+ is selective for shape as well as for motion [34]. In spite of that, we did not observe decoding of motion direction from V5/hMT+.

It is interesting that our main analysis did not replicate the result of Kamitani & Tong [15] in decoding motion from visual cortex activity using classifiers trained on motion (faster or slower). Our classifier analyses replicated only the ability to classify static-oriented stimuli [14,16]. There are several possible reasons for this. First, as pointed out earlier, previous work has used hard-edged-moving dot stimuli, which are spatially broadband and would have produced streaks over a wide range of spatial frequencies. By contrast, here we used Gaussian blob stimuli, which are spatially narrowband. Thus, if streak information were essential for successful decoding, previously used motion stimuli would contain streaks over a much wider range of spatial scales than our narrowband Gaussian blobs, which might have provided more streak information for decoding. Second, because receptive field sizes for motion are larger than those for orientation [35], and motion receptive fields are also estimated to be larger for lower spatial frequencies [36], it is possible that motion information for these relatively low spatial frequencies was not available at a large enough scale. Similarly, although we replicated previous studies [14,16] showing significant decoding of static orientation stimuli from V1 and V2, we did not observe significantly above-chance decoding of orientation from V3. This may also be due to differences in the stimuli used; previous work used broadband square-wave gratings as opposed to the relatively narrowband filtered noise patterns we used here. This may have reduced the orientation-selective signal somewhat when compared with previous experiments and may have biased our results towards V1 and V2. Importantly, the successful decoding of motion from purely static-oriented stimuli that we observed was qualitatively similar across different classification methods (figure 5 and electronic supplementary material, figure S3), survived reversal of generalization direction (training on motion and decoding orientation) and thus provides clear evidence for motion streaks. The absence of significant decoding of motion alone thus may also indicate that the motion streak signal itself was very weak; it is only possible to reliably generalize between ‘streaky’ motion and static-oriented stimuli.

Our findings show that motion streaks are speed-dependent, raising the intriguing possibility that earlier claims for successful decoding of motion direction from patterns of brain activity [15,30] might in fact rely on motion streaks. In these studies, dots moved at speeds above the streak threshold [1,10]. Both studies also found much better decoding in early visual cortex than in V5/hMT+, which was attributed to the lower number of voxels in V5/hMT+. Our findings raise the alternative possibility that superior decoding in early visual areas might result from decoding of motion streaks activating orientation-selective neurons in these areas. Interestingly, in Kamitani & Tong's [15] second experiment (decoding attended direction), which used rotating motion stimuli where motion streaks would not have been informative, the level of decoding in V5/hMT+ was much higher relative to earlier areas. No previous decoding study has used motion below 2° per second, although previous studies reported robust BOLD signals to motions at these speeds [37] and we also observed this in our study (figure 4). Thus, the difficulty in decoding slow motion in our study might reflect the fact that previous successful motion decoding relied on motion streaks. Future studies investigating classification of motion in fMRI data should be careful to separate the effect of motion streaks on classification from that of mechanisms more traditionally associated with motion perception.

It should be noted that, although we refer throughout to ‘fast’ and ‘slow’ motions, indicating motion that is either above or below the critical speed for motion streaks established in previous psychophysical work, this is to some extent an arbitrary dichotomy. It is likely that the tuning of neurons to motion direction and orientation varies with speed; this notion is supported by optical imaging and modelling studies [5,6]. Moreover, the generation of ‘streaky’ motion depends not just on tuning but on the temporal response profile of the small neuronal circuits involved in processing motion in retinotopic cortices and MT. There is therefore likely to be some form of monotonic relationship between the speed of a stimulus and the magnitude of streak signals it produces. Future studies could therefore investigate this relationship by examining whether generalization from motion direction to orientation varied parametrically with motion speed, and the form of such a relationship.

It is important to note that even if the classifier had exploited another aspect of visually evoked brain activity than orientation-selectivity—for instance, radial bias [38,39]—it is the *orientation* signal that must be relevant for generalizing between static and moving stimuli, whether this is a fine-grained signal or a relatively coarse pattern [40]. Any bias due to motion would make comparisons between different directions of motion more likely to generalize from fast to slow motion than from static orientation to motion. We did not find generalization from fast to slow or from slow to fast motion in any of the visual areas, which is surprising in the light of previous models of motion perception. Some studies report separate channels for high-speed and low-speed motion [41–43], but an alternative possibility is that the high-speed channel might combine information from static, oriented signals with those from opponent signals for direction. This is consistent with Geisler's original hypothesis that there are separate systems for faster and slower motions. It is possible that more effective motion stimuli, such as drifting gratings, or more spatially broadband dot stimuli, could generalize between slower and faster motions.

We speculate that motion too slow to form streaks may be processed by a system more akin to the classical model of motion perception, where neurons sensitive to a particular direction of motion are also most sensitive to orientations orthogonal to their preferred direction [26–28]; this is consistent with our psychophysical results. Conversely, as motion becomes fast enough for streaks to provide a useful source of information, the sustained orientation signal formed by streaks excites neurons sensitive to orientations parallel to the motion trajectory [1,3,5,6,44]. Our work has implications for models of motion perception, both in understanding human vision and in creating computer vision systems for motion recognition [45]. In particular, it is possible that the information from the two systems might be combined with a weighting relative to their reliability, similar to the Bayesian framework used for multisensory integration [46,47]; this remains to be investigated.

Overall, our results provide support for the motion streak hypothesis and represent the first evidence for the neural representation of streaks in human early visual cortex.
